# Personalized Network Mapping Reveals State-Trait Dissociation of Frontoparietal Connectivity in Adolescent Depression: A Longitudinal Study

**DOI:** 10.21203/rs.3.rs-11393774/v1

**Published:** 2026-10-20

**Authors:** Jasper Laca, Amar Ojha, Megan M. Herting, Anurima Mummaneni, Arielle Keller, Lucina Uddin, Tiffany Ho

## Abstract

Prior research linking resting-state networks (RSNs) to adolescent depression has yielded inconsistent findings, partly because group-average templates poorly capture individual brains; a mismatch that may be especially consequential during adolescence, when RSNs grow increasingly heterogeneous across individuals. Using a personalized analysis pipeline in the longitudinal Characterizing Adolescent Trajectories of Depression cohort (38 depressed adolescents [28 female]; ages 14–18; 181 usable scans; avg. participation: 446.18 days), we derived individualized definitions of three canonical depression-related RSNs. Because longitudinal RSN connectivity may reflect both trait-like vulnerability and state-like symptom fluctuation, we used mixed-effects models to decompose connectivity profiles into both components. FPN connectivity showed a significant state-trait dissociation: chronically elevated connectivity predicted greater average symptom burden, while transient increases predicted momentary relief. These effects were undetectable using group-atlas estimates. We suggest FPN connectivity plays a dual, compensatory role in adolescent depression and demonstrate that personalized definitions can reveal clinically meaningful brain–symptom associations.

